# Characteristics of Non-mass Enhancement in Contrast-enhanced Breast MRI and Associations with Malignancy

**DOI:** 10.7150/jca.114087

**Published:** 2025-07-04

**Authors:** Kamber Goksu, Ahmet Vural, Ahmet Nedim Kahraman

**Affiliations:** University of Health Sciences Fatih Sultan Mehmet Training and Research Hospital, Department of Radiology, Istanbul, TURKIYE.

**Keywords:** breast cancer, magnetic resonance imaging (MRI), non-mass enhancement, diagnosis

## Abstract

**Background:** Magnetic resonance imaging (MRI) has a limited role in distinguishing non-mass enhancement (NME) lesions as benign or malignant and determining whether the lesions are invasive or not. In this study, we aimed to investigate the differences in MRI of benign and malignant NME lesions and to determine the relationship between the pattern of enhancement in NME lesions and histopathologic diagnosis.

**Materials and methods:** Breast MRI examinations (n=5214) performed at the study institution between January 2018 and July 2024 were evaluated. We enrolled 460 patients in the study. NME lesions were classified according to the BI-RADS atlas. In addition, linear enhancements were divided into branching and non-branching. Factors showing significant associations in univariate analyses were evaluated with multivariate analyses using the logistic regression model. The assessments were performed by two radiologists who are experienced in breast imaging.

**Results:** This study included 460 NME lesions (342 benign and 118 malignant). Focal and segmental distribution, dynamic enhancement features, Type I (persistent) and Type III (wash-out) dynamic curve modes, and clustered-ring internal enhancement pattern features showed statistically significant differences in terms of differentiating benign from malignant (P<0.05). Heterogeneous enhancement gave significant results in distinguishing invasive carcinoma from ductal carcinoma in situ (DCIS) (P<0.05). Wash-out type curve from dynamic enhancement curves was also seen at a higher rate in invasive carcinomas. Although the general results are similar to previous studies, in our study, unlike other studies, enhancements showing linear distribution were divided into two groups branching and non-branching, and lesion size was measured. It was observed that branching enhancements and lesion sizes greater than 15 mm significantly indicated malignancy (p<0.05).

**Conclusions:** MRI is a valuable way to identify malignant NME lesions and may be useful in determining whether the lesions are invasive or not. Evaluating NME lesions with breast MRI can help decide on biopsy when branching types of lesions with linear distribution and lesions greater than 15 mm are detected.

## Introduction

**Figure 1:**
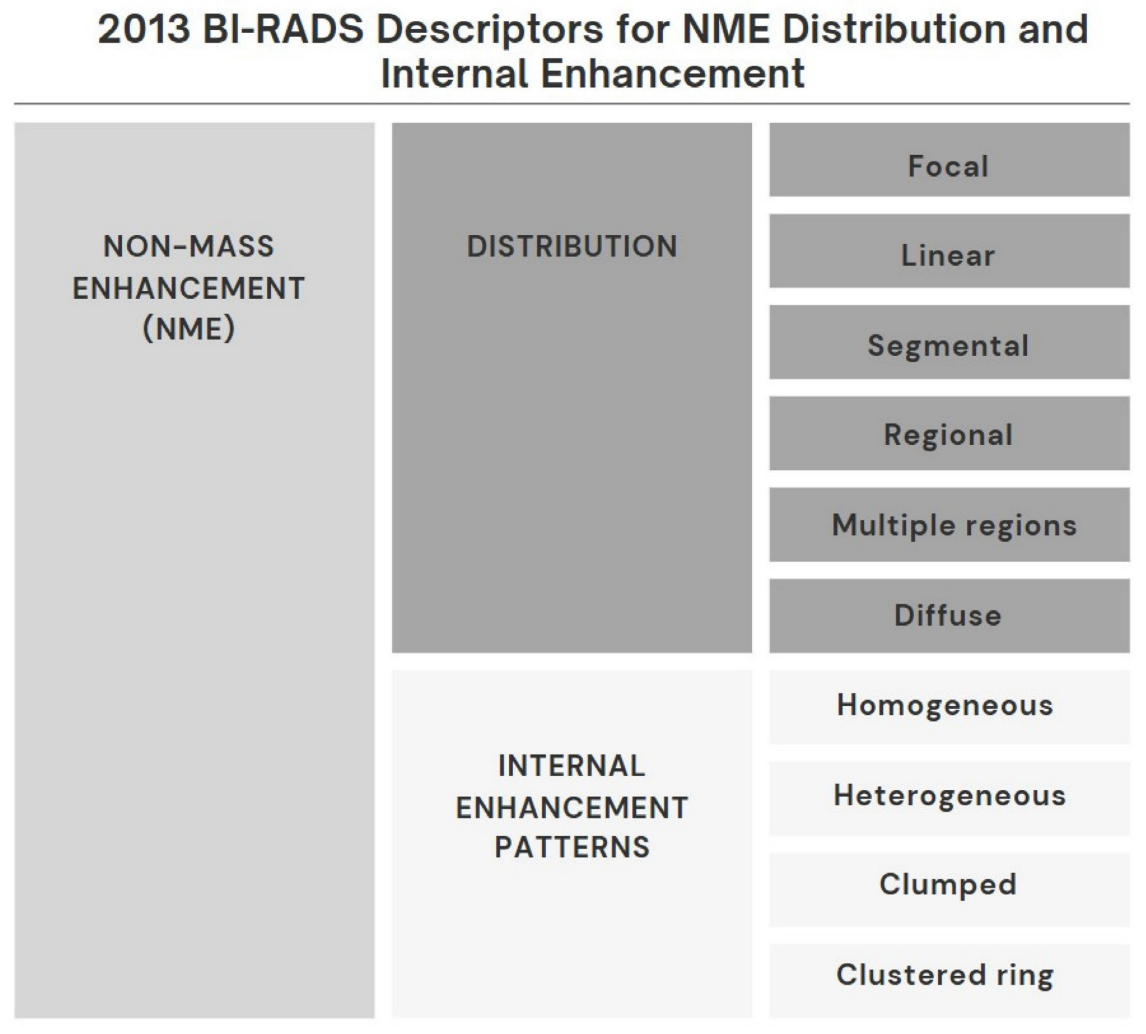
BI-RADS descriptors for NME lesions.

Breast magnetic resonance imaging (MRI) is used in breast imaging for the detection of breast cancer, characterization of the lesion, preoperative evaluation, strategy determination for the lesion detected in the breast, and evaluation of response to treatment due to its high diagnostic accuracy 1. MR imaging findings are very important in distinguishing lesions that can be followed from lesions that require biopsy 1. In recent years, diagnostic methods based on the Breast Imaging Reporting and Data System (BI-RADS) published by the American College of Radiology have been used. Lesions are classified as mass, non-mass enhancement (NME), or focus 2,3. BI-RADS defines NME as a small or large area of enhancement that is not a mass or focus and can be distinguished from Background Parenchymal Enhancement (BPE) 3. A mass is a 3-dimensional space-occupying lesion with convex borders. A focus can be defined as a contrasting spot smaller than 5 mm that is too small to be characterized morphologically and has no corresponding findings on pre-contrast images 3. Any non-BPE enhancement that does not meet these criteria is classified as NME. Some BI-RADS descriptors for the distribution and enhancement patterns of NME are more suspicious for malignancy than others (Figure 1). NME seen on breast MR imaging can represent a variety of benign breast abnormalities, as well as invasive carcinomas and ductal carcinoma in situ (DCIS) 4-7. There is ongoing debate about whether lesions classified as BI-RADS category 3 in particular require follow-up or biopsy. Recent studies have shown that MR imaging is increasingly playing a role in making this decision. Despite this, there is little data to support decisions about which lesions should be followed and which lesions should be sent for biopsy 8-10.

## Materials and Methods

### Patients

The study was conducted with a protocol in accordance with the Declaration of Helsinki and was conducted retrospectively after receiving ethics committee approval (permission document numbered 2021/34). 5214 breast MRI studies performed in our institution be-tween January 2018 and July 2024 were evaluated. Cases with NME lesions detected in breast MRI were identified with the study we conducted in our database.

For inclusion in the study, cases were identified in which a final diagnosis was made by tissue sampling or in which the stability or regression of findings could be confirmed. For NME lesions without histopathological data, they were considered benign if the lesion disappeared in at least one follow-up study or if the lesion remained stable for at least 2 years.

The inclusion criteria for the study were as follows: Presence of NME on MRI. Breast lesions with pathological results and clinical data. Exclusion criteria were as follows: Previously treated lesions. Any problems that could affect the evaluation in imaging (incomplete sequence, inadequate image quality, etc.). As a result, 460 patients were included in the study and the pathology of NME lesions was diagnosed using excision biopsy or US-guided core needle biopsy. The 460 lesions included 118 malignant NME lesions (90 DCIS, 28 invasive carcinoma) and 342 benign NME lesions (84 intraductal papilloma, 114 adenosis-fibrosis, 55 fibroadenoma, 48 lesions without inflammation or nonspecific neoplasia, 41 lesions that were stable or disappeared during follow-up) (Table 1).

### MRI imaging technique

MRI was performed using a 1.5T MRI unit (Optima MR450w, GE Healthcare) with a dedicated 8-channel chest coil in the prone position. Multiparametric MRI images of the breast were obtained, including T1- and T2-weighted, diffusion-weighted, and dynamic contrast-enhanced images. Typical MRI parameters used are detailed in Table 2. Gadopentetate dimeglumine (Magnevist; Bayer Healthcare, Wuppertal, Germany) was automatically injected as a contrast agent at a rate of 2.0 mL/s. The contrast dose (0.1 mmol/kg) was determined according to the patient's weight. The slice thickness was 2 mm without an intersection gap (Table 2).

Both breasts were examined in the axial plane on six-phase dynamic images obtained 30 seconds, 1 minute, 1.5 minutes, 2 minutes, 2.5 minutes, and 3 minutes after contrast medium injection, respectively. Additionally, bilateral sagittal fat-suppressed T2-weighted images and coronal diffusion-weighted images were obtained before contrast medium administration.

### Interpretation of MRI images and data acquisition

All breast MR images were initially interpreted by a radiologist (AV, with 20 years of experience in breast imaging). This radiologist was informed about the patient's clinical in-formation and imaging findings, including those obtained from mammography and breast ultrasonography. Image interpretation of NME was performed according to the latest edition of the BI-RADS MR imaging dictionary [3) (Figure 1). The largest dimension of the NME was measured.

For each breast MRI examination, the initial interpretations were recorded in the database. A second radiologist (KG, with 22 years of experience in breast imaging) then retrospectively reviewed the database of interpretations made by the first radiologist, blinded to the interpretation of the first radiologist, and analysed the findings. In cases where there could be considered a difference of opinion, both radiologists evaluated together, and the joint evaluation decisions were accepted as the final result. If a patient had more than one lesion, the lesion with the highest BI-RADS category was considered as the data and included in the analyses.

### Statistical analysis

All statistical analyses were performed using SPSS, version 18.0 (SPSS, Chicago, IL, USA), and P<0.05 was considered as the threshold for statistically significant difference. Pearson χ2 test or Fisher's exact test was used to determine the differences in MRI features between benign and malignant breast lesions. The receiver operating characteristic (ROC) curve was used to evaluate the diagnostic values of these features. Positive predictive values (PPVs) and 95% confidence intervals of malignancy were calculated and compared ac-cording to lesion size and subtype of linear enhancement. Lesion size and subtype of non-mass enhancement used were taken from the data obtained from the interpretation made by both radiologists as a consensus. Student t test was performed for the analysis of between-group differences in continuous variables. Factors showing a significant association with the outcome in univariate analysis were then evaluated by multivariate analysis using the logistic regression model.

## Results

During the study period, we retrospectively evaluated 5214 cases that underwent breast MRI examination. Of these patients, 1416 cases with NME lesions on breast MRI images were detected. When the data of these cases were evaluated, 956 cases that had previously undergone any treatment, had no pathology results, had problems that could affect the evaluation on imaging, and had inadequate follow-up were excluded from the study. The mean age of the 460 patients included in the study was 49.5±7.2 years (range, 29-78). The mean body weight of the patients was 70.5±8 kg (range, 51-121). When the patients were divided into two groups according to median age (under and over 50 years), the malignancy rates did not show a significant difference between younger and older patients (P =0.544).

Of the 460 patients with NME lesions, 118 had malignant lesions (90 DCIS, 28 invasive carcinoma) and 342 had benign lesions (114 adenosis-fibrosis, 55 fibroadenoma, 84 intraductal papilloma, 48 inflammation or nonspecific diagnoses without neoplasia, 41 stable or disappearing lesions during follow-up).

**Figure 2:**
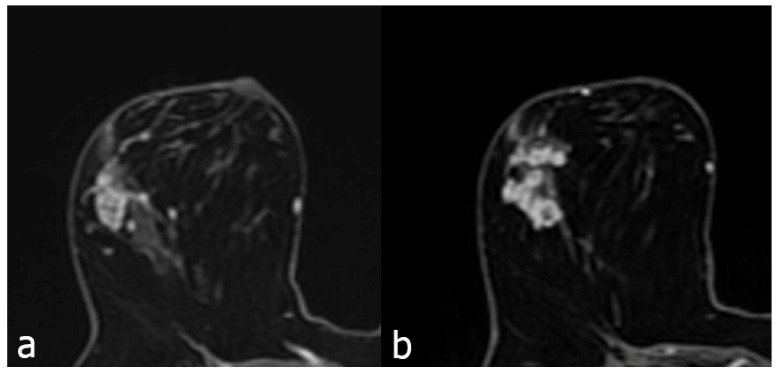
Contrast enhanced breast MRI, focal heterogeneous enhancing NME lesion (a), biopsy result of DCIS in a 45-year-old patient. Second image (b), 52 years old, focal clustered-ring enhancing NME lesion, invasive carcinoma as a result of biopsy.

**Figure 3:**
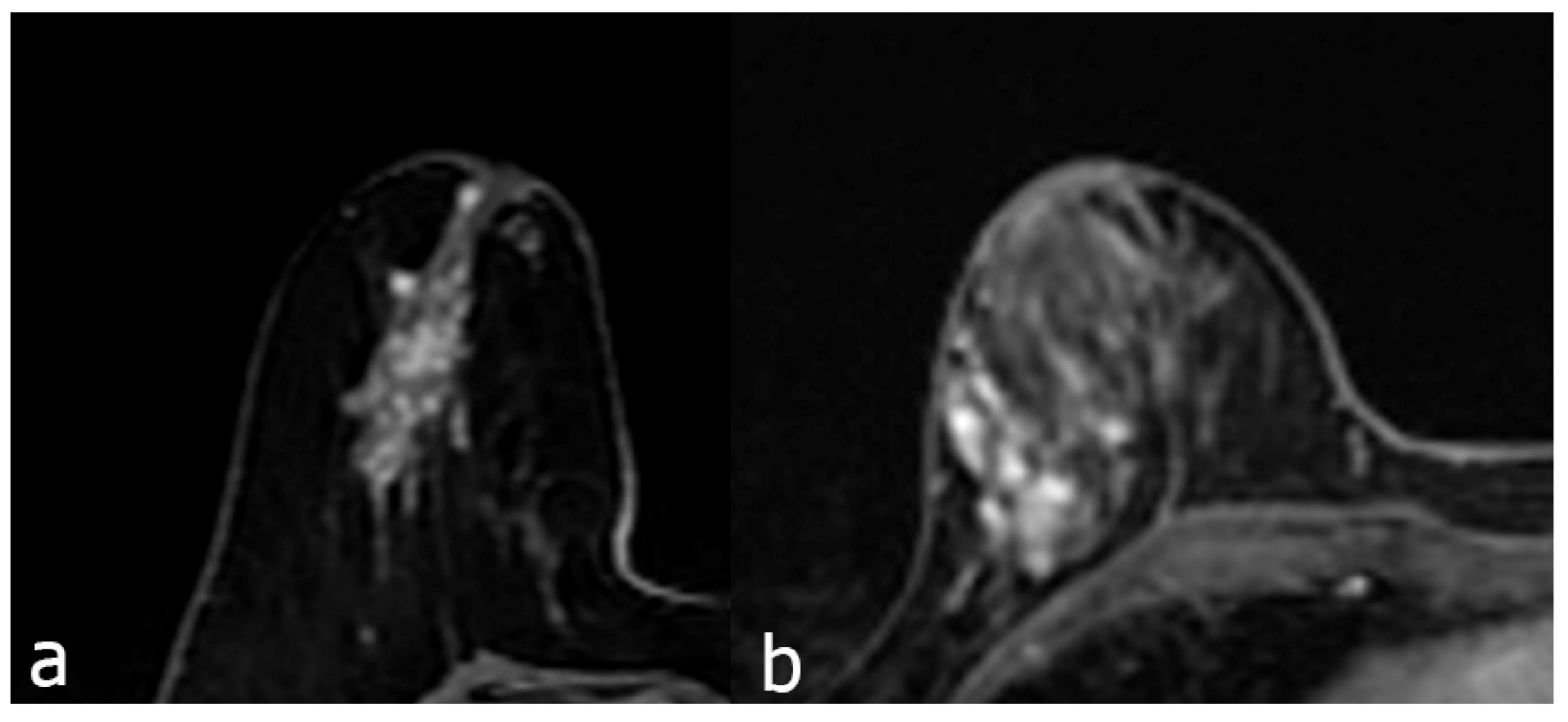
The first contrast enhanced breast MRI image (a), 49 years old, is a segmental heterogeneous enhancing NME lesion, and the pathology result is adenosis. Second image (b), 41 years old, regional heterogeneous enhancing NME lesion, biopsy result is invasive carcinoma.

The contrast enhancement characteristics of patients with NME lesions detected on MR imaging are summarized in Table 3. Among the contrast distribution characteristics of NME breast lesions, there was a statistically significant difference between benign and malignant lesions in those showing linear, segmental, and regional distribution (P<0.05). Segmental distribution was higher in malignant NME lesions (35/118, 29.7%) than in segmental distribution in benign lesions (71/342, 20.8%). While 82 (24%) of benign NME lesions showed linear distribution, 41 (34.7%) of malignant lesions showed linear distribution. In those showing regional distribution, the proportion of malignant lesions (8/118, 6.8%) was found to be benign while the proportion of lesions (56/342, 16.4%) was found to be benign. Among internal enhancement patterns, the frequency of clustered-ring enhancement was statistically significantly higher in malignancies (20/118, 16.9%) than in benign lesions (33/342, 9.6%) (P<0.05). In those with heterogeneous internal enhancement patterns, benign NME lesions (182/342, 53.2%) were more common than malignant lesions (47/118, 39.8%). The difference between benign and malignant NME lesions for persistent type (type I) and wash-out type curve (type III) from dynamic enhancement curves was statistically significant (P<0.05) (Figure 2 and 3). The wash-out type curve (type III) was more common in malignancy (63/118, 53.4%) and higher than in the benign group (109/342, 31.9%). The incidence of type I was higher in benign lesions (99/342, 28.9%) than in malignant lesions (8/118, 6.8%) (P=0.001). The sensitivity and specificity of the kinetic curve model in predicting malignant NME lesions were 93.2% and 71.1%. In terms of diffusion restriction, diffusion restriction was observed in 94 (79.7%) of malignant lesions, while diffusion restriction was observed in 244 (71.3%) of benign lesions, which was not statistically significant (P=0.178) (Table 3).

**Figure 4:**
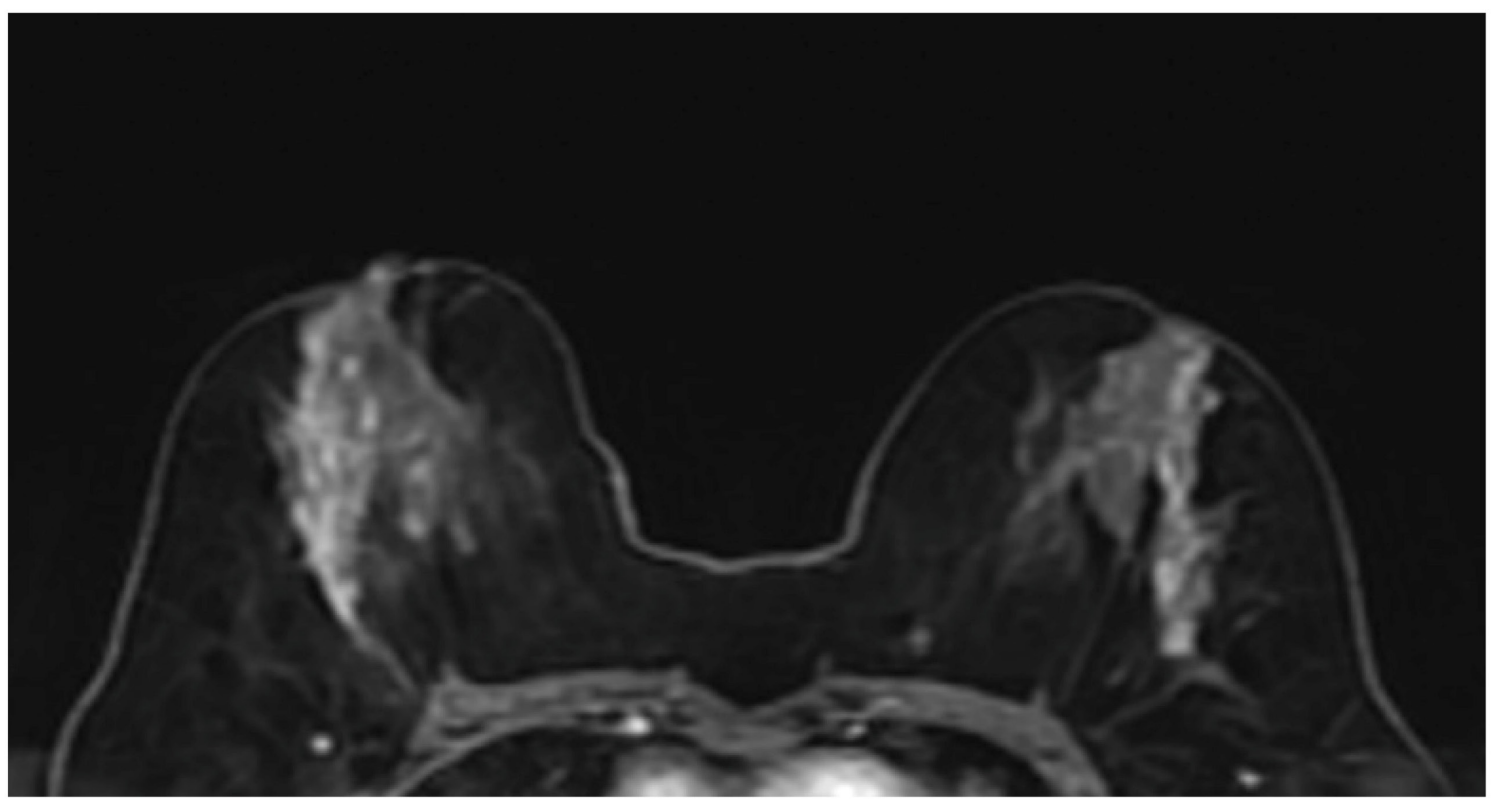
MRI image showed segmental heterogeneous enhancing NME lesion on the right and linear heterogeneous enhancing NME lesion on the left in a 46-year-old patient. Right breast biopsy result: DCIS, left breast biopsy result: atypical ductal hyperplasia.

The MRI features of malignant lesions were further analysed and the results are shown in Table 4. The MRI features of DCIS and invasive cancer were compared with benign lesions. Among the distribution features and internal enhancement patterns, linear, segmental, regional distribution, and clustered-ring enhancement can help distinguish between benign and malignant NME lesions (Figure 4). However, there was no statistical difference in the distribution features between invasive carcinoma and carcinoma in situ. Interestingly, in terms of internal enhancement patterns, the rates of heterogeneous enhancement for invasive carcinoma (4/28, 14.3%) were found to be statistically significantly lower than those for DCIS lesions (43/90, 47.8%) (P<0.05). The rates of clumped and clustered-ring enhancement were found to be higher in invasive carcinoma (11/28, 39.3% and 9/28, 32.1%, respectively) than in DCIS lesions (21/90, 23.3% and 11/90, 12.2%, respectively) (P<0.05). According to dynamic curves, benign lesions were more likely to show type I features in both groups (P=0.001, 0.009). The wash-out curve (type III) was higher in both invasive cancer (14/28, 75%) and DCIS (49/90, 54.4%) than in benign lesions (109/342, 31.9%) (P<0.05). Twenty-three (82.1%) of invasive cancers showed diffusion restriction, while 244 (71.3%) of benign lesions showed diffusion restriction. The difference was statistically significant (P<0.05). However, there was no significant difference in diffusion restriction between DCIS and benign lesions.

**Figure 5:**
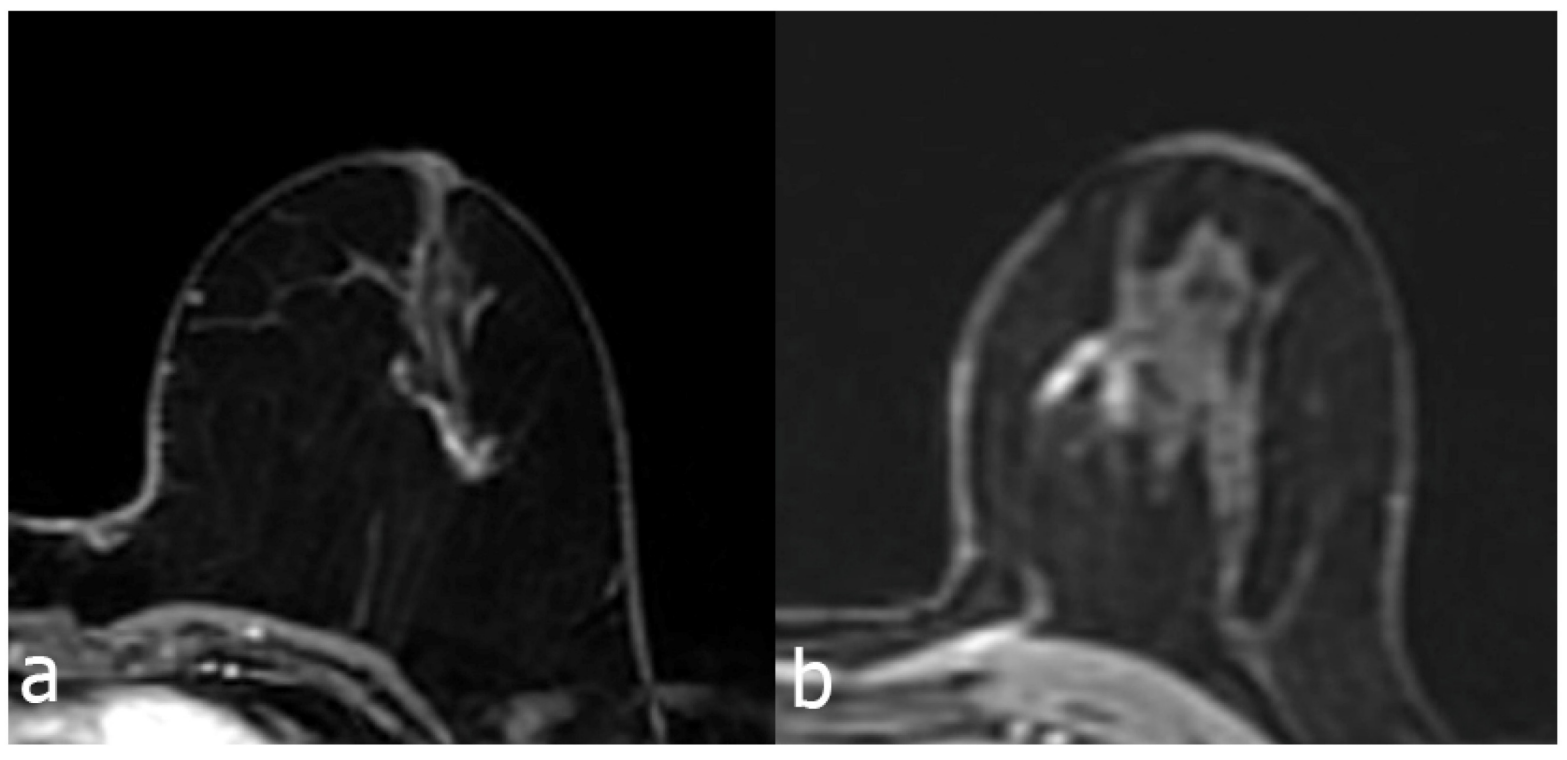
Contrast enhanced breast MRI, linear heterogeneous branching type enhancing NME lesion (a) 56 years old, atypical ductal hyperplasia due to biopsy. Similarly, in a 44-year-old patient with NME lesion showing linear heterogeneous branching contrast enhancement (b), the biopsy result was DCIS.

The 123 cases showing linear distribution were additionally divided into two groups according to whether the contrast enhancement showed branching or not. Of the cases showing linear distribution, 75 did not show branching, while 48 showed branching. Of the cases showing branching, 22 (45.8%) were malignant, and 26 (54.2%) were benign pathologies. Of the cases not branching, 19 (25.4%) were malignant, and 56 (74.6) were benign pathologies (Figure 5). When malignant pathologies were evaluated as DCIS and invasive carcinoma; 16 of the branching group (n=22) were DCIS and 6 were invasive carcinoma. In the non-branching malignant group (n=19), 17 cases were DCIS and 2 cases were invasive carcinoma (Table 5).

The lengths of NME lesions showing linear distribution were divided into two groups (less than 15 mm and over 15 mm) and evaluated. For lesions shorter than 15 mm, the number of benign lesions was significantly higher than the number of malignant lesions (P<0.05). While the benign status of lesions without branching was higher than the malignant status, the malignant rate was higher than the benign rate in branching lesions (p<0.005).

The PPV of the non-branching pattern was 25% (19 of 75 lesions; 95% CI: 20-31%). In contrast, the PPV of the branching pattern was 46% (22 of 48 lesions; 95% CI: 40-51%). The PPV of the branching pattern was significantly higher than the PPV of the non-branching pattern (P<0.005).

The PPV of lesions <15 mm was 27% (23 of 84 lesions; 95% CI: 23-32%). In contrast, the PPV of lesions >15 mm was 46% (18 of 39 lesions; 95% CI: 41-57%). The PPV of lesions >15 mm was higher than that of lesions <15 mm (P<0.05).

In univariate analysis, the two subtypes of linear distribution (branching or non-branching) and lesion size (less than 15 mm and over 15 mm) in NME lesions were significantly associated with pathology results in terms of malignancy and benign status in both groups when evaluated separately. In multivariate analysis, both factors were found to be significant predictors of malignancy (Table 6).

## Discussion

NME lesions detected on dynamic contrast-enhanced breast MRI have attracted increasing attention in recent years 11-14. Recent studies on NME lesions have provided valuable information on differentiating benign and malignant lesions and on biopsy decisions 15-17. In their study, Asada et al. 18 reported that segmental distribution was significantly associated with malignancy; in this study, the malignancy rate was found to be significantly higher in NME lesions with segmental distribution (35/118, 29.7% in malignant lesions) (P<0.05). We also found that linear distribution was similarly more frequent in malignant lesions (41/118, 34.7%) and that this was higher than in the benign NME group (82/342, 24%), and the difference was statistically significant (P<0.05). We evaluated the cases with linear distribution in two groups as branching and non-branching, and al-so measured the longest dimension of the lesions with linear distribution. In this study, the PPV of cases showing branching was significantly higher than the PPV of cases showing no branching (P<0.005). In addition, the PPV of lesions greater than 15 mm was higher than the PPV of lesions less than 15 mm (P<0.05). Previously reported PPVs of NME lesions showing linear distribution ranged from 9% to 67% 4-6,19,20. These findings suggest that NME lesions showing linear distribution may include more than one lesion type with different PPVs; therefore, it may be more accurate to evaluate NME lesions showing linear distribution in more than one group. Tozaki and Fukuda 4 reported a significant difference in the PPVs for malignancy between those showing branching and those showing no branching in their interpretation of the model for interpreting NME lesions with some modifications. Both of these patterns are categorized as linear distribution in the current BI-RADS classification. In our study, the PPV of the branching group was statistically significantly higher than the PPV of the non-branching group (P<0.05). In addition, the PPV of lesions greater than 15 mm was found to be significantly higher than the PPV of lesions less than 15 mm (P<0.05). We believe that future changes to the current BI-RADS classification could include an additional assessment of branching and lesion size for linearly distributed NMEs. Our findings suggest that lesions that do not show branching and are smaller than 15 mm in size are more likely to be benign, whereas lesions that show branching and are greater than 15 mm are more likely to be malignant. Based on our findings, it can be decided that NME lesions less than 15 mm and showing a non-branching linear distribution can be followed up, and that a biopsy is required if a linear distribution NME lesion shows branching or is greater than 15 mm. Although the possibility of malignity cannot be completely ruled out for lesions detected with a non-branching linear distribution and less than 15 mm, follow-up examination may be sufficient at least until there is a change in the shape or size of the NME lesion.

In previous years, Gutierrez et al. 21 reported that MRI findings were not significant predictors of malignancy in NME lesions detected on breast MRI but not detected on mammography and examination. In our study, we found that some internal enhancement pat-tern findings, together with the distribution, had the potential to predict malignancy. Regarding internal enhancement patterns, previous studies reported that clustered-ring enhancement could be successful in detecting malignant NME lesions 12. It was found that NME lesions with heterogeneous enhancement (malignant 47/118, 39.8%; benign 182/342, 53.2%) were more common in benign lesions (P<0.05). In NME lesions with clustered-ring enhancement (malignant 20/118, 16.9%; benign 33/342, 9.6%), the rate of malignancy was significantly higher (P<0.05). In addition, when invasive carcinoma (invasive 9/28, 32.1%; benign 33/342, 9.6%) and DCIS (DCIS 11/90, 12.2%; benign 33/342, 9.6%) were evaluated separately for clustered-ring enhancement, it was noted that the rate of invasive carcinoma was higher. Clustered-ring enhancement may be an important predictor of malignancy of NME lesions, and this result is consistent with the findings of Lunkiewicz 13. Machida et al. evaluated 76 DCIS and 55 invasive breast cancers presenting as NME and found that clustered-ring enhancement was significantly associated with invasion 14. Hahn et al. also reported that clustered-ring enhancement was more common in microinvasive ductal carcinoma than in pure DCIS 22.

Although the role of dynamic enhancement curve in mass lesions has been known for a long time, there are studies reporting that it is less effective for NME lesions than for mass lesions 23. In this study, malignant NME lesions generally showed a washout type curve (Type 3) and benign NME lesions generally showed a persistent (Type 1) dynamic curve, which was statistically significant (P<0.05). Malignant NME lesions were then divided into two groups (invasive carcinoma and DCIS), and invasive breast cancer of NME mostly showed both Type 2 and Type 3 more than benign lesions, which was statistically significant (P<0.05). Similar results were obtained for DCIS. However, no statistically significant difference was found in dynamic enhancement for the distinction between DCIS and invasive carcinoma. Therefore, dynamic curves may help distinguish malignant NME breast cancer from benign lesions, but they do not effectively distinguish invasive carcinoma from DCIS. Greenwood et al. They reported that morphological features were more effective than dynamic enhancement in determining DCIS in MRI evaluation 24. As a result, it seems that a washout curve (Type 3) similar to mass lesions would be useful in identifying malignant NME lesions.

In this study, we did not measure the diffusion rate or ADC value. However, we evaluated whether the lesion we defined showed diffusion restriction. The diffusion restriction rate in malignant lesions (94/118, 79.7%) was higher than in benign lesions (244/342, 71.3%), but the difference was not statistically significant (P=0.178). However, diffusion restriction was found to be significantly higher in invasive carcinomas (23/28, 82.1%) than in benign lesions (244/342, 71.3%) (P<0.05). Another study reported that the diffusion restriction rate was significantly higher in malignant NME lesions compared to benign lesions due to in-creased cell density 25. The differences in results may be due to different ROI measurement methods and different technical parameters in different studies. In addition, measuring the DWI ratios and ADC value (b = 1,000 s/mm2) and comparing them with benign lesions for invasive cancer or DCIS will provide more detailed information. Greenwood et al. reported that DWI and ADC values can distinguish DCIS from invasive disease, and that invasive cancer has a lower mean ADC value than DCIS (P<0.01) 24.

There are some limitations to our study. First of all, although the measurements and interpretations were confirmed by a second radiologist in case of a possible suspicion, they were mainly based on the evaluation of a single radiologist. In addition, the radiologist who made the evaluation was aware of the assumption that NME lesions with a linear distribution and branching structure were more likely to be malignant 4,26,27. In addition, a pool of patients who were diagnosed and followed up in our clinic was used. Therefore, although an attempt was made to evaluate by ignoring the diagnoses of the patients, being familiar with the diagnoses of the patients may have affected the evaluation. This may have led to a selection bias. In addition, diffusion restriction was subjectively evaluated as present or absent. DWI and ADC values were not measured. The metabolic status of the patients, menopause status, and possible drug and hormone usage status related to these conditions were not evaluated. The effects of such drugs and hormonal therapeutics on cancer development and background enhancement that hides lesions are known 28-30. Lesions that disappeared during follow-up, although not many, or those that regressed during the 2-year follow-up were considered benign, and the lack of pathological evaluation of these patients can be considered another limitation of our study. All results obtained in the current study are population-based, and the results of this study were not evaluated by comparing them with a completely normal control group. The retrospective nature of this study and the limited sample size can be considered as limitations, and further studies with larger sample sizes are needed to confirm the results. In addition, false negative results may occur in the pathological results of percutaneous biopsy, which may affect the accuracy of our results.

In conclusion, this study has shown that MRI is useful in distinguishing malignant NME lesions. Segmental distribution, clustered-ring enhancement, and wash-out dynamic enhancement kinetics have been associated with malignancy. On the other hand, in lesions with linear distribution, non-branching, and/or smaller than 15 mm, Type I persistent dynamic enhancement curve are also seen to be predictive of benign lesions. Breast MRI is a valuable method for predicting the probability of malignancy and invasiveness of NME lesions.

## Figures and Tables

**Table 1:** Pathologic category of NME lesions

Category	No (%)
	
Malignant	118 (25,7)
DCIS	90 (19,6)
Invasive carcinoma	28 (6,1)
	
Benign	342 (74,3)
Intraductal papilloma	84 (18,3)
Adenosis- Fibrosis	114 (24,8)
Fibroadenoma	55 (11,9)
Inflammation- nonspecific	48 (10,4)
Stable or disappeared	41 (8,9)
	
Total	460 (%100)

**Table 2:** Imaging parameters for breast MR examination

Parameter	T1-weighted MR Imaging	T2-weighted MR Imaging	DW MR Imaging	DCE MR Imaging
Repetition time (msec)/ Echo time (msec)	8.3/4.7	3200-3500/90-100	7000-8000/80-90	3.9/1.9
Matrix	420x440 mm	204x256	128x128	320x320
Flip angle (degrees)	25	90	90	15
Section thickness (mm)	5	5	2,4	2,4
Field of view (mm)	360	360	320	320
No. of signals acquired	2	2	2	NA
b value (sec/mm2)	NA	NA	0/800	NA
Temporal resolution (sec)	NA	NA	NA	60

**Table 3:** MR imaging findings in NME lesions

MRI Findings				
	Malignant (n=118) (%)	Benign (n=342) (%)	P value	Total (n=460) (%)
Distribution				
Focal	24 (20.3)	98 (28.7)	0.078	122 (26.5)
Linear	41 (34.7)	82 (24)	0.023*	123 (26.7)
Segmental	35 (29.7)	71 (20.8)	0.048*	106 (23)
Regional	8 (6.8)	56 (16.4)	0.009*	64 (13.9)
Multiple Regions	5 (4.2)	14 (4.1)	0.946	19 (4.1)
Diffuse	5 (4.2)	21 (6.1)	0.440	26 (5.7)
Internal enhancement patterns				
Homogeneous	19 (16.1)	58 (17)	0.998	77 (16.7)
Heterogeneous	47 (39.8)	182 (53.2)	0.012*	229 (49.8)
Clumped	32 (27.1)	69 (20.1)	0.116	101 (22)
Clustered-ring	20 (16.9)	33 (9.6)	0.045*	53 (11.5)
Dynamic curve			0.001*	
Persistent - Type I	8 (6.8)	99 (28.9)		107 (23.3)
Plateau - Type II	47 (39.8)	134 (39.2)		181 (39.3)
Wash-out - Type III	63 (53.4)	109 (31.9)		172 (37.4)
Diffusion restriction			0.178	
Present	94 (79.7)	244 (71.3)		338 (73.5)
Absent	24 (21.3)	98 (28.7)		122 (26.5)

*, P<0.05. NME = non-mass enhancement, MRI = magnetic resonance imaging

**Table 4:** MRI findings between pathological malignant NME lesions and benign lesions

	Invasive Carcinoma (n=28) (%)	Benign (n=342) (%)	P value	DCIS (n=90) (%)	Benign (n=342) (%)	P value
Distribution						
Focal	5 (17.9)	98 (28.7)	0.199	19 (21.1)	98 (28.7)	0.078
Linear	8 (28.6)	82 (24)	0.072	33 (36.7)	82 (24)	0.052
Segmental	8 (28.6)	71 (20.8)	0.139	27 (30)	71 (20.8)	0.075
Regional	3 (10.7)	56 (16.4)	0.027	5 (5.6)	56 (16.4)	0.031
Multiple Regions	2 (7.1)	14 (4.1)	0.696	3 (3.3)	14 (4.1)	0.675
Diffuse	2 (7.1)	21 (6.1)	0.555	3 (3.3)	21 (6.1)	0.664
Internal enhancement patterns						
Homogeneous	4 (14.3)	58 (17)	0.989	15 (16.7)	58 (17)	0.959
Heterogeneous	4 (14.3)	182 (53.2)	0.001	43 (47.8)	182 (53.2)	0.004
Clumped	11 (39.3)	69 (20.2)	0.034	21 (23.3)	69 (20.2)	0.075
Clustered-ring	9 (32.1)	33 (9.6)	0.006	11 (12.2)	33 (9.6)	0.028
Dynamic curve			0.001			0.109
Persistent - Type I	0 (0)	99 (28.9)		8 (8.9)	99 (28.9)	
Plateau - Type II	14 (50)	134 (39.2)		33 (36.7)	134 (39.2)	
Wash-out - Type III	14 (50)	109 (31.9)		49 (54.4)	109 (31.9)	
Diffusion restriction			0.043			0.185
Present	23 (82.1)	244 (71.3)		71 (78.9)	244 (71.3)	
Absent	5 (17.9)	98 (28.7)		19 (21.1)	98 (28.7)	

NME = non-mass enhancement, MRI = magnetic resonance imaging, DCIS = Ductal carcinoma *in situ*

**Table 5:** Pathologic categories of Linear Distribution NME lesions

Pathology	Non-branching (n=75)	Branching (n=48)
	(%)	(%)
Malignant		
DCIS	17 (22.7)	16 (33.3)
İnvasive carcinoma	2 (2.7)	6 (12.5)
Total	19 (25.4)	22 (45.8)
Benign		
All benign results	56 (74.6)	26 (54.2)

NME = non-mass enhancement, DCIS = Ductal carcinoma *in situ*

**Table 6:** PPVs by branching and size in Linear Distributed NME lesions

	Benign	Malignant	PPVs (%)	P value
Findings				
Non-branching	56	19	25.3 (19.8- 30.8)	0.020
Branching	26	22	45.8 (40.2- 51.2)	
Less than 15 mm	61	23	27.4 (22.9- 31.9)	0.045
Greater than 15 mm	21	18	46.2 (40.7- 51.7)	
Results of Multivariate Analysis				
Factor	Odds Ratio*			P value
Branching compared non-branching	25.1 (13.6- 80.1)			0.019
Less than 15mm vs Greater than 15mm	20.2 (11.2- 65.5)			0.087

* Numbers in parentheses are 95% Cıs, PPV = positive predictive values
